# A Multispectral Image Creating Method for a New Airborne Four-Camera System with Different Bandpass Filters

**DOI:** 10.3390/s150717453

**Published:** 2015-07-20

**Authors:** Hanlun Li, Aiwu Zhang, Shaoxing Hu

**Affiliations:** 1 Key Laboratory of 3D Information Acquisition and Application of Ministry, Capital Normal University, Beijing 100048, China; E-Mail: lihanlun@126.com; 2 School of Mechanical Engineering & Automation, Beihang University, Beijing 100083, China; E-Mail: husx@buaa.edu.cn

**Keywords:** panchromatic camera, filters, SIFT, RANSAC, registration, multispectral

## Abstract

This paper describes an airborne high resolution four-camera multispectral system which mainly consists of four identical monochrome cameras equipped with four interchangeable bandpass filters. For this multispectral system, an automatic multispectral data composing method was proposed. The homography registration model was chosen, and the scale-invariant feature transform (SIFT) and random sample consensus (RANSAC) were used to generate matching points. For the difficult registration problem between visible band images and near-infrared band images in cases lacking manmade objects, we presented an effective method based on the structural characteristics of the system. Experiments show that our method can acquire high quality multispectral images and the band-to-band alignment error of the composed multiple spectral images is less than 2.5 pixels.

## 1. Introduction

**Figure 1:**
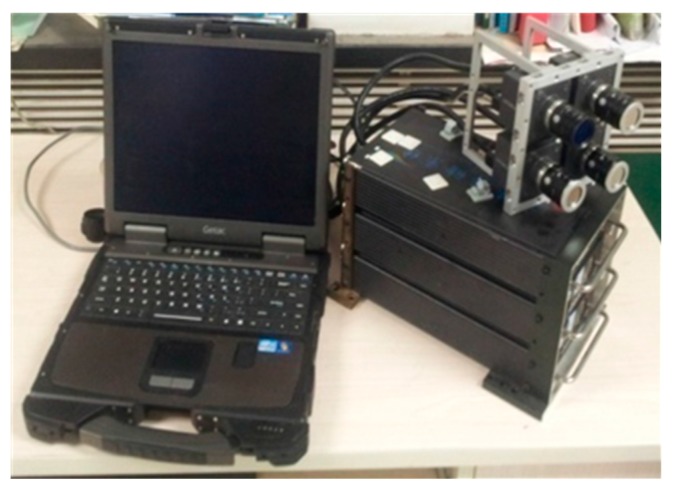
The four-camera multispectral mapping system.

With the rapid development of unmanned aerial vehicle (UAV) technology, we urgently need a low cost multispectral system which can acquire multispectral images at the wavelengths based on actual requirements. Our research group developed an airborne high resolution multispectral system (Figure 1) which is mainly composed of a set of digital video recorders (DVR), a ruggedized Getac B300 PC, four identical Hitachi KPF120CL monochrome cameras (2/3 inch Interline type, Progressive Scan CCD), and four bandpass filters. The four identical monochrome cameras are sensitive in the 400 to 1000 nm spectral range, have the capability of obtaining 8-bit images with 1392 × 1040 pixels, and are respectively equipped with near-infrared (800 nm), red (650 nm), green (550 nm) and blue (450 nm) bandpass filters. As a result, it has the flexibility to change filters to acquire other band images in the 400 to 1000 nm spectral range for specific requirements. Because the four cameras are independent, it has the advantage that each camera can be individually adjusted for optimum focus and aperture setting. However, for the multiple optical systems, it is nearly impossible to align different band images taken by the cameras at one exposal optically or mechanically [1], so a registration method is needed.

In recent years, many multispectral mapping systems were developed. For example, a multispectral image system called MS4100 (Geospatial Systems, Inc., West Henrietta, NY, USA) used a beam splitting prism and three charge coupled device (CCD) sensors to acquire images in three spectral bands within the 400–1100 nm. Although the alignment issue may become easier, it is difficult to change the band-pass filters once integrated. Oppelt [2] introduced an imaging system. As with the MS4100, once it is integrated, the band images at other wavelengths cannot be acquired. A complex imaging system equipped with a lens, a cold mirror, a beamsplitter, three rear lens units, three filters and three monochrome cameras was introduced by Kise [3]. It is not easy to change filters and hard to extend to get other band images at different wavelengths. Everitt [4] proposed a multispectral digital video system which is comprised of three video CCD analog video cameras and three filters. Its hardware design and video data synchronization acquisition were introduced in detail. Gorsevski [5] designed an airborne mapping system that can provide valuable experiential learning opportunities for students. It is a very complex system, and the multispectral system is only one of its subsystems. Most of these four papers focus on hardware design and data synchronization acquisition but fail to introduce alignment methods in detail. Honkavaara [6] introduced a complex weight UAV spectral camera. This spectral camera acquires different band images by changing its air gap. The dislocation between different bands depends on flight speed and flying height. Due to different structures, its band matching methods are not very suitable for our multispectral system. Yang [7,8] used first-order and second-order polynomial transformation models to register band images and successfully obtained multispectral images. However, the polynomial model is just a generic registration model; the structural characteristics of the system itself were not fully considered, so the transformation may not be modeled properly. In addition, the method to generate matching points has not been introduced.

Due to great altitude and limited undulating ground, this paper considers the land surface as a plane and has proved that the homography registration model is very suitable. Currently, many papers [9,10,11,12,13] use SIFT [14,15] and RANSAC [16] to get the parameters of transformation models. The RANSAC algorithm is a learning technique to estimate parameters of a specific model by random sampling of observed data and uses a voting scheme to find the optimal fitting result; the voting scheme is based on an assumption that there are enough inliers (correct matches in this paper) to satisfy the specific model. When the number of inliers is less than 50% [17], it usually performs badly. However, the different band images acquired by our system differ from each other in the image intensity, and the correct rate of initial matches declines rapidly. Especially for pairs including an infrared band image and a visible band image, the correct rate is even less than 10% if the images comprise a considerable part of vegetation and very few manmade objects. So, the RANSAC cannot be used directly. Very few papers focused on registering pairs of infrared images and visible images. In light of this, according to the structural characteristics of the four-camera multispectral system, this paper proposed an effective method to remove most of the false matches and greatly increase the correct rate, and then uses the RANSAC to eliminate the remaining false matches. Finally, parameters of the registration model are calculated using the least squares method. Experiments show that this method does not only improve the registration performance, but also solves the matching problem between near-infrared images and visible images in the case of lack of manmade objects.

This article developed an airborne high resolution four-camera multispectral system, proposed an excellent registration method and introduces this method in detail. The second part describes the derivation of the matching model and its parameters calculation method; the third part introduces the method of eliminating false matches; the fourth part shows the different band image registration experiments; the fifth part shows the data acquisition and accuracy assessment; and the final part gives a conclusion.

## 2. Registration Model and Parameters Calculation

Because the four cameras are independent, transformation relationships among different band images must be known before the process of multispectral image composition. The derivation of the transformation model and the parameters calculation method are as follows.

### 2.1. The Derivation of the Transformation Model

The multispectral system consists of four cameras, and their IDs respectively are 0, 1, 2, and 3. For any point *W* in the three dimensional space, its coordinate is $W=[X_{W}\;\;Y_{W}\;Z_{W}]$ in matrix form, ${\left[\begin{array}{cc} W & 1 \end{array}\right]}^{T}$ in homogeneous coordinates. $v={\left[x\;\;\;y\;\;\;\;\;-f\right]}^{T}$ denotes its corresponding image/space point. According to the pinhole imaging principle [1], the relationship between **W** and **v** can be described as Equation (1):

(1) $$\lambda v=\left[\begin{array}{cc} K & 0 \end{array}\right]\left[\begin{array}{cc} R & t\\ 0^{T} & 1 \end{array}\right]{\left[\begin{array}{cc} W & 1 \end{array}\right]}^{T}={\text{KRW}}^{T}+\text{Kt}$$

**K**, **R** and **t** are a 3 × 3 camera calibration matrix, 3 × 3 rotation matrix, and 3 × 1 translation vector respectively. **v_i_** is the corresponding image/space point in the *i*-th camera. When World Coordinate System (WCS) coincides with the Camera Coordinate System (CCS) of the 0-th camera, we obtain:

(2) $$\lambda_{0}v_{0}=K_{0}\text{IW}+K0=K_{0}W$$

(3) $$\lambda_{i}v_{i}=K_{i}R_{i}W+K_{i}t_{i}$$

Due to the great altitude, small field angle, limited undulating ground, and negligible offsets, the land surface is considered as a plane. Assuming that the normal vector of the plane is **m**, **m**^*T*^**W** = *N*, and *N* is a constant, Equation (3) can be written as Equation (4):

(4) $$\lambda_{i}v_{i}=K_{i}R_{i}W+K_{i}t_{i}\frac{m^{T}W}{N}$$

According to Equations (2) and (4), we obtain:

(5) $$v_{i}=\frac{\lambda_{0}}{\lambda_{i}}K_{i}(R_{i}+\frac{t_{i}m^{T}}{N})K_{0}^{-1}v_{0}=H_{i}v_{0}$$

**H_i_**, the so-called homography, is a 3 × 3 matrix determined by the scale factors λ_0_, λ_*i*_, calibration matrix **K_0_**, **K_i_**, normal vector **m**, rotation matrix **R_i_**, and translation vector **t_i_**; so it can model the transformation relationship between the images properly.

### 2.2. Transformation Model Parameters Calculation

As a 3 × 3 matrix, **H_i_** has nine unknown variables, eight of which are independent. In total, it has eight degrees of freedom; every pair of matched points can build two equations, so we need four matches to solve the **H_i_**. However, the noise and the other errors lessen credibility for the lack of the rest observations when we only have four pairs of matching points. To calculate the **H_i_**, we need more than four matches for adjustment so as to promote credibility and accuracy. Equation (5) can be written as Equation (6):

(6) $$\left[\begin{array}{c} x_{i}\\ y_{i}\\ 1 \end{array}\right]=\left[\begin{array}{ccc} h_{11}^{i} & h_{12}^{i} & h_{13}^{i}\\ h_{21}^{i} & h_{22}^{i} & h_{23}^{i}\\ h_{31}^{i} & h_{32}^{i} & h_{33}^{i} \end{array}\right]\left[\begin{array}{c} x_{0}\\ y_{0}\\ 1 \end{array}\right]$$

For any feature point (*x*_0_, *y*_0_) in the image taken by the 0-th camera, where (*x_i_*, *y_i_*) is its matching point taken by the *i*-th camera, the error equation can be described as Equation (7):

(7) $$\varepsilon_{xi}=x_{i}-\frac{h_{11}^{i}x_{0}+h_{12}^{i}y_{0}+h_{13}^{i}}{h_{31}^{i}x_{0}+h_{32}^{i}y_{0}+h_{33}^{i}},\;\varepsilon_{yi}=y_{i}-\frac{h_{21}^{i}x_{0}+h_{22}^{i}y_{0}+h_{23}^{i}}{h_{31}^{i}x_{0}+h_{32}^{i}y_{0}+h_{33}^{i}}$$

In order to get the **H_i_**, we just need to use the least square method to minimize the ε_*i*_. The process of multispectral image composition is as follows: three empty output matrixes are created at the same pixel size as the reference image. For any one of the three, using the transformation model **H_i_**, the coordinates of each pixel in the output image matrix are transformed to determine their corresponding location in the original input image. However, because of small differences in the CCD sensors and orientations of the cameras in the aluminum frame, this transformed cell location will not directly overlay a pixel in the input matrix. Interpolation is used to assign a Digital Number (DN) value to the output matrix pixel determined on the basis of the pixel values that surround its transformed position in the input image matrix. So far, there are some common interpolation methods can be chosen, including nearest neighbor algorithm, bilinear interpolation, cubic convolution, and Inverse Distance Weighted (IDW). The nearest neighbor algorithm is adopted because it simply assigns the closest pixel value in the input image to the new pixel and does not change the original data values. Of course, we may use other complex interpolation methods if necessary.

## 3. Rejecting False Matches

Image feature points are extracted by the SIFT detector. The SIFT feature is a kind of local feature of digital images using 128-dimensional vectors to describe feature points. It maintains invariance of scaling and rotation and also keeps a certain degree of stability with change of brightness, the viewing change, affine transformation and noise. It consists of four major stages: (1) scale-space peak selection; (2) keypoint localization; (3) orientation assignment; and (4) keypoint descriptor. There are a lot of false matches in the initial matches, so the SIFT initial matches cannot be used to calculate the parameters of the transformation model directly. The conventional approach is to apply RANSAC to eliminate false matches. The RANSAC method is based on the random sampling theory and requires the correct rate of initial matches higher than 50%. In general, the correct rate of initial matches can satisfy the requirement of the RANSAC, such as a pair of adjacent aerial images. However, owing to the four monochrome cameras equipped with different bandpass filters lead to the significant difference in the image intensity among different band images. Thus the number of incorrect matches will increase rapidly and the correct rate will decline sharply, especially for pairs of infrared and visible images. If the image is full of vegetation, the variation of the intensity between pairs of infrared and visible images is more significant. In this case, the correct rate is very low, and the RANSAC is not reliable.

In light of this situation, based on the structural characteristics of the multispectral system, the paper presents an effective method to eliminate most of the false matches and to improve the correct rate for meeting the requirement of the RANSAC. The principle of the method is as follows.

**Figure 2:**
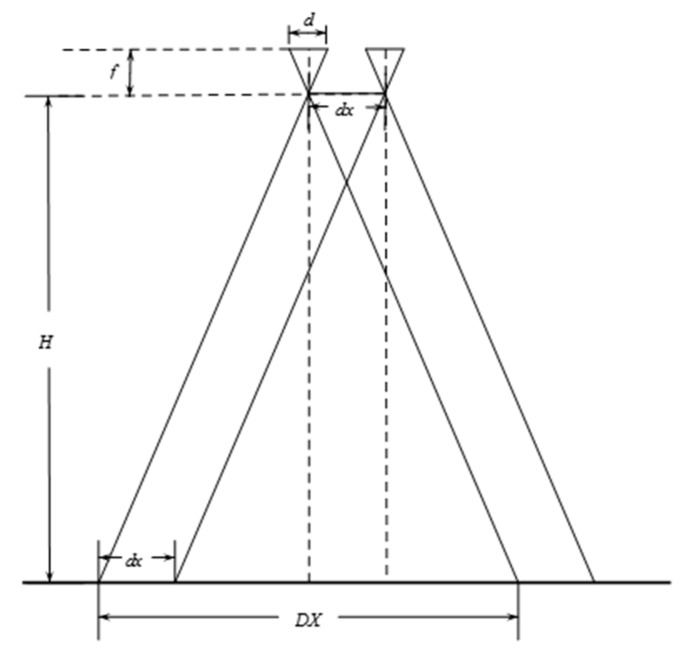
Schematic diagram of two camera imaging.

Figure 2 shows two cameras of the four-camera system, the flying altitude *H*, the focal length *f*, and the length of CCD *d*. The field of a camera can be described as Equation (8):

(8) $$DX=H\frac{d}{f}$$

If the CCD has *M* cells, the ground resolution can be calculated as Equation (9):

(9) $$R=\frac{DX}{M}=\frac{Hd}{Mf}$$

*dx* indicates the distance between the optical centers of the two cameras. If these two cameras are the same and parallel, the displacement between the fields of the two cameras also is *dx*. The displacement between the two images taken by the two cameras in *X* direction is *xcol*, as Equation (10):

(10) $$xcol=\frac{dx}{R}=\frac{dxMf}{Hd}$$

*dx* is 0.136 m, and *M* is 1040 in *X* direction; we could know that *f/d* is 2.376 after calibrating the camera intrinsic parameters using the Camera Calibration Toolbox [18]. To ensure the safety of the aircraft, we need the flying height to be large enough, at least 50 m, then *xrow* is 5.74 pixels. Assuming that the height change of ground is less than 10 m, the stereo parallax will be less than 1.16 pixels. Therefore, the stereo effect can be ignored. Using the same method, we can also calculate the displacement in *Y* direction *yrow*.

**Figure 3:**
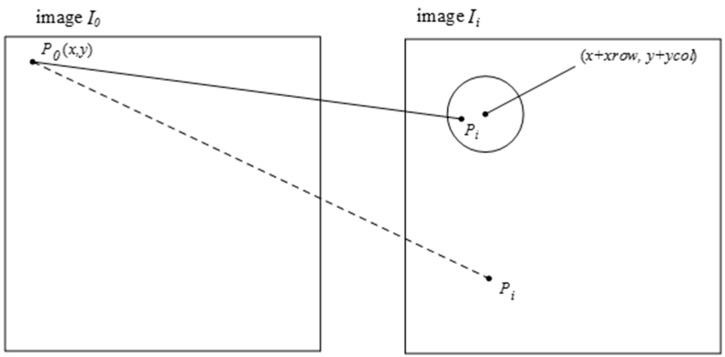
The dotted line shows a false match because *p_i_* is too far away from (*x + xrow*, *y + ycol*). And, the match showed by the solid line will be remained.

If the flying height is fixed, there are no changes in flight attitude and no ups and downs on the ground, the camera plane parallels to the ground surface, the cameras are arrangement in parallel, there is no camera lenses distortion and CCD distortion, then the displacement (*xrow*, *ycol*) between the two images is fixed. If all of the *dx*, *M*, *d*, *H* and *f* are known, the (*xrow*, *ycol*) can be calculated by using Equation (10) directly. However, if not all of these parameters are known, we need other methods. An effective histogram method is proposed, and we will describe it in Section 4. For any feature point *p*_0_ with coordinate (*x*, *y*) in reference image *I*_0_, the coordinate of its matching point *p_i_* is (*x + xrow*, *y + ycol*) in input image *I_i_*. Although these ideal situations do not occur in actuality, the influence of all these factors is limited for our multispectral system because the arrangement of the four cameras is almost parallel, as Figure 1. So, it can be estimated that *p_i_* is near (*x + xrow*, *y + ycol*) in the input image *I_i_*. We just need to set a threshold to check whether *p_i_* is near (*x + xrow*, *y + ycol*). If the threshold is too low, some correct matches can be rejected. And, if it is too high, the removed false matches will decrease. In this paper, the threshold is set to one tenth of the image size, 104 pixels. If the Euclidean distance between *p_i_* and (*x + xrow*, *y + ycol*) is lower than this threshold, the match will be retained in this step, described as the solid line in Figure 3; otherwise, it will be taken as a false matching point and be eliminated, described as the dotted line in Figure 3. Due to this, the correct rate of initial matches will increase significantly and the RANSAC approach will become more reliable.

## 4. Different Band Image Registration

### 4.1. Experiment 1

**Figure 4:**
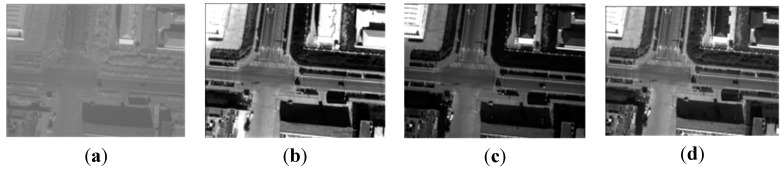
The band images; (**a**) the infrared band; (**b**) the red band; (**c**) the green band; (**d**) the blue band.

**Figure 5:**
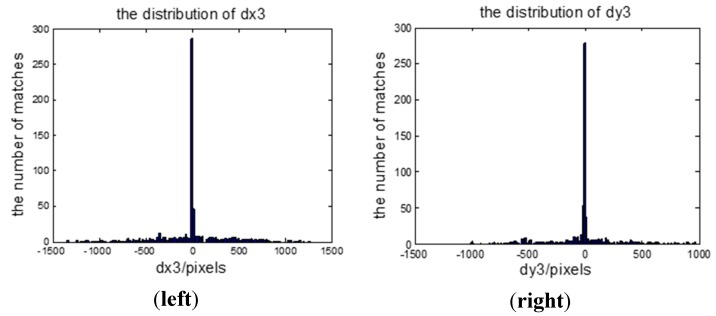
The matches’ coordinate displacements; (**left**) the distribution of *dx*_3_; (**right**) the distribution of *dy*_3_.

We select four synchronous images acquired by our multispectral system, which contain many artificial objects, as shown in Figure 4. The number of SIFT feature points of the four images respectively is 5097, 6481, 5911, and 6618. Because green vegetation has higher reflection levels in the infrared and green bands, and lower reflection levels in the red and blue bands, green bands are more similar to infrared bands in vegetation areas, and the registration between the green band and the other two visible bands is easier, so the green band is chosen as the reference band image, and the other three will be registered to it. The total initial matches respectively are 1446, 1139, and 485. For all initial matches, the coordinates of the key points in the reference band image are respectively (*x*_01_, *y*_01_), (*x*_02_, *y*_02_), …, (*x*_0*n*_, *y*_0*n*_) where the coordinates of the corresponding points in the input band image are respectively (*x*_*i*1_, *y*_*i*1_), (*x*_*i*2_, *y*_*i*2_), …, (*x_in_*, *y_in_*), and *i* = 1, 2, 3 represents the points in the other three bands. Letting *d_xi_* = *x*_0_ – *x_i_*, *d_yi_* = *y*_0_ – *y_i_*, *d*_*x*3_ and *d*_*y*3_ respectively indicates the matches’ coordinate displacements between the green band image and the infrared band image in X direction and Y direction. To represent distribution of *d*_*x*3_ and *d*_*y*3_, two histograms have been created, as shown in Figure 5. We can see that the number of matches reaches the top when *d*_*x*3_ is at −6.17; the number of matches reaches a maximum when *d*_*y*3_ is at −8.84. It can be estimated that (*xrow*, *ycol*) is approximately equal to (−6.17, −8.84) for the green band and infrared band pair. We can use the same method to calculate the displacements between the green band and other two bands. If flying height and flying attitude vary a little, the evaluated (−6.17, −8.84) can be used to register other images taken at other times. The correct matches respectively are 1278, 973, and 257, with the correct rate of 88%, 85% and 53%. The correct rate of initial matches between the green band and the infrared band is significantly lower than the correct rates between the green band and the other two bands. However, because of many manmade objects, all the correct rates are higher than 50%, so we can still use the RANSAC method to eliminate the error matches directly.

### 4.2. Experiment 2

**Figure 6:**
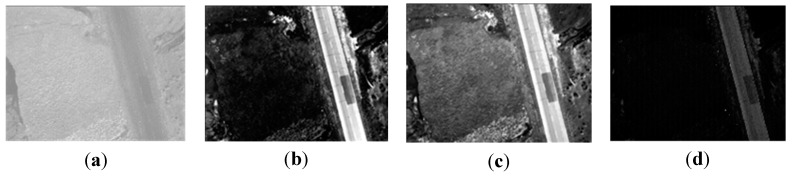
The band images with less artificial objects; (**a**) the infrared band; (**b**) the red band; (**c**) the green band; (**d**) the blue band.

**Figure 7:**
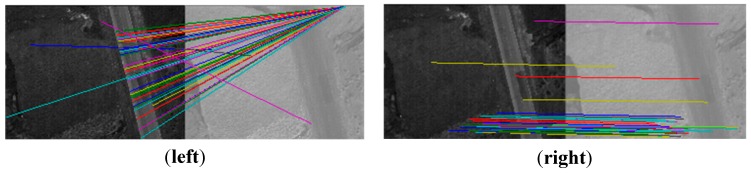
Image registration; (**left**) the result obtained by using the RANSAC directly; (**right**) the result obtained by using our method.

As shown in Figure 6, there are four different band images acquired by the multispectral system at one exposure. Compared with Figure 4, the four pictures shown in Figure 6 contain fewer artificial objects. The number of SIFT feature points in the four images respectively is 4739, 7964, 7497 and 8999. For the same reason as in Experiment 1, the green band is chosen to be the reference band image, and the other three band images are registered to it. As shown in Table 1, the initial matches (IM) respectively are 1446, 1139, 537, and the correct matches (CM) respectively are 1038, 926, 36; the correct rate (CR) respectively is 72%, 85%, 7%. The correct rate (CR) between the green band and the infrared band is significantly lower than 50%. It is unable to get the correct result using the RANSAC directly, as shown in Figure 7 (left). Because Figure 6 and Figure 4 are from a same flight strip, there is little change in flying height and flying attitude. So, the evaluated (*xrow*, *ycol*) in Experiment 1 can be used to eliminate false matches. 398, 185, and 491 false matches have respectively been removed, as shown in Table 1. After eliminating, the matches respectively become 1048, 954, 46, and the correct rates respectively become 99%, 97%, 78% , so the registration performance is significantly improved. All of these are much higher than 50%, making RANSAC more reliable, and solving the registration problem between the green band and infrared band, as shown in Figure 7 (right).

**Table 1:** The number of matches and correct rate.

Image Pairs	Experiment 2					Experiment 3				
	IM	CM	CR (%)	RFM	CR (%)	IM	CM	CR (%)	RFM	CR (%)
G-B	1446	1038	72	398	99	555	342	61	210	99
G-R	1139	926	85	185	97	792	525	66	255	98
G-IR	537	36	7	491	78	303	19	6	277	73

### 4.3. Experiment 3

**Figure 8:**
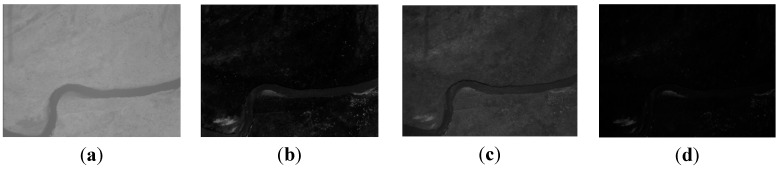
The band images with no artificial objects; (**a**) the infrared band; (**b**) the red band; (**c**) the green band; (**d**) the blue band.

**Figure 9:**
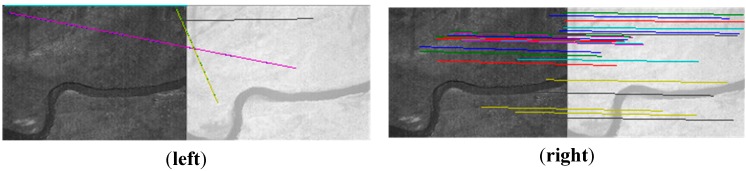
Image registration; (**left**) the result obtained by using the RANSAC directly; (**right**) the result obtained by using our method.

As shown in Figure 8, four different band images acquired by the multispectral system at one other exposal are selected. Compared with Figure 4 and Figure 6, Figure 8 contains no artificial objects. The number of SIFT feature points in the four images are 4137, 5578, 5469, and 7385, respectively. For the same reason as in the above two experiments, the green band is chosen as the reference band, and the other three band images are registered to it. As shown in Table 1, the numbers of initial matches (IM) are 555, 792 and 303, and the correct matches (CM) respectively are 342, 525 and 19, thus the correct rate (CR) respectively is 61%, 66% and 6%. The correct rate between the green band and the infrared band is evidently lower than 50%. Therefore the RANSAC cannot be used directly, as shown in Figure 9 (left). Because Figure 4, Figure 6 and Figure 8 are from a same flight strip, they have little change in flying height and attitude. So, the evaluated (*xrow*, *ycol*) in Experiment 1 can also be used to eliminate error matches in this experiment. As shown in Table 1, 210, 255 and 277 false matches (RFM) have been eliminated respectively. After that, the number of matches respectively become 345, 537 and 26, and the correct rates (CR) respectively becomes 99%, 97%, 73%; the registration performance is significantly improved. All of these percentages are much higher than 50%, and the RANSAC is more reliable and can be used directly to obtain correct matching results, as shown in Figure 9 (right).

## 5. Four-Band Multispectral Data Acquisition and Accuracy Assessment

**Figure 10:**
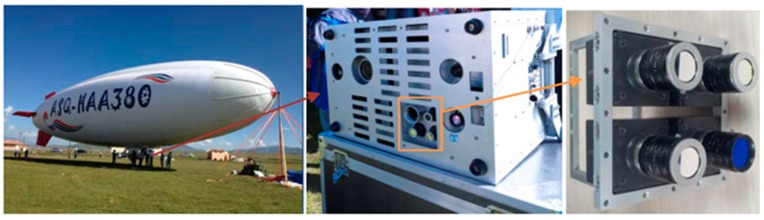
Experimental scene and equipment installation.

**Figure 11:**
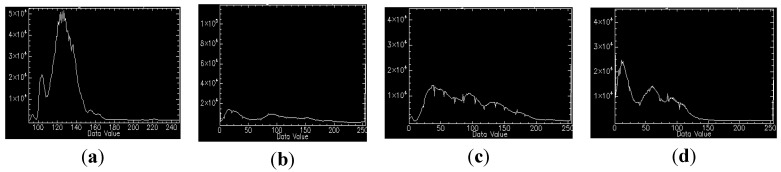
Histograms of the four band images; (**a**–**d**) respectively the histograms of the infrared band, red band, green band and blue band.

The multispectral mapping system was mounted on a metal protective box installed on an airship, named ASQ-HAA380, which was developed by our research group. On 18 August 2014, the research group carried a flight experiment in Haibei Tibetan Autonomous Prefecture, Qinghai Province, China, and the experimental scenario is shown in Figure 10. Qinghai TV and many other media sources reported this experiment [19]. The experimental data will be used mainly for pasture biomass assessment and the survey of urban green space in high altitude area. In order to guarantee the image quality of each multispectral camera, according to flight altitude and weather conditions, each camera was individually adjusted for optimum focus and aperture setting. Four different band images acquired by the multispectral system at one exposal are shown in Figure 4 in the Section 3, and their histograms are shown in Figure 11. The histograms of these images from diverse target areas spread well within the dynamic range without saturation and indicate that the system is able to capture high quality multispectral data.

### 5.1. Experiment 1

**Figure 12:**
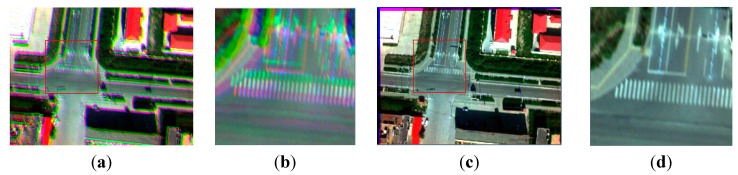
The true-color composite (red, green, blue); (**a**) the unregistered multispectral image; (**b**) the enlarged partial region of (**a**); (**c**) the registered multispectral image; (**d**) the enlarged partial region of (**c**).

**Figure 13:**
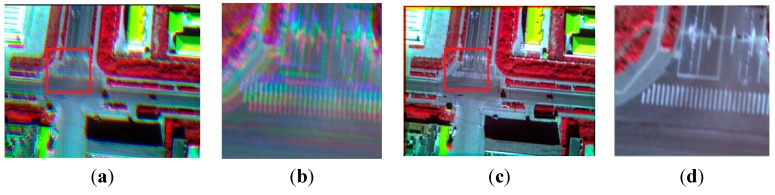
The CIR composite (infrared, red, green); (**a**) the unregistered multispectral image; (**b**) the enlarged partial region of (**a**); (**c**) the registered multispectral image; (**d**) the enlarged partial region of (**c**).

In this experiment, four images containing a lot of man-made objects are used, as shown in Figure 4. Because it contains a lot of man-made objects, the SIFT operator can extract enough effective feature points, and the correct rate is higher than 50%, so the RANSAC can be used directly. Using the homography transformation model and the nearest neighbor interpolation method mapping the blue band, the red band, and the near infrared band to the green band, a four-band multispectral image is obtained. Figure 12 and Figure 13 depict the true-color composite and the CIR composite of the four-band image respectively. Figure 12a and Figure 13a depict the unregistered multispectral image, and Figure 12b and Figure 13b, respectively, display their enlarged partial regions. Figure 12c and Figure 13c depict the registered multispectral images, and Figure 12d and Figure 13d displays their enlarged partial regions. There are severe dislocations between different bands of the unregistered multispectral image. In contrast, these dislocations disappear in the registered multispectral image.

The difference among the results of the four methods is in subpixel level, unable to be recognized by the naked eye. In order to have a quantitative measure of it objectively, an inverse transformation is performed using the inverse matrix of *H_i_*, which can transform the coordinates of the input image to the reference coordinates. For any input point (*x_i_*, *y_i_*), its retransformed point (*x_r_*, *y_r_*) in the reference image can be derived from Equation (6) by replacing (*x*_0_, *y*_0_) with (*x_r_*, *y_r_*), shown in Equation (11):

(11) $$\left[\begin{array}{c} x_{r}\\ y_{r}\\ 1 \end{array}\right]={\left[\begin{array}{ccc} h_{11}^{i} & h_{12}^{i} & h_{13}^{i}\\ h_{21}^{i} & h_{22}^{i} & h_{23}^{i}\\ h_{31}^{i} & h_{32}^{i} & h_{33}^{i} \end{array}\right]}^{-1}\left[\begin{array}{c} x_{i}\\ y_{i}\\ 1 \end{array}\right]$$

The residual, *XR*, is the difference between *x*_0_ and *x_r_*, and *YR* is the difference between *y*_0_ and *y_r_*. The root mean square error, *R*, is the distance between the reference point and the retransformed point in the reference image coordinate system. *XR*, *YR* and *R*, for any match are calculated with distance formulas:

(12) $$XR=\sqrt{{\left(x_{r}-x_{0}\right)}^{2}},\;YR=\sqrt{{\left(y_{r}-y_{0}\right)}^{2}},\;R=\sqrt{{XR}^{2}+{YR}^{2}}$$

with *n*, as the amount of the point pairs, *j*, as the serial number, *R_x_*, the root mean square error in *x* direction, *R_y_*, the root mean square error in *y* direction, *R_t_*, the total root mean square error, can be calculated as the following formulas:

(13) $$R_{y}=\sqrt{\frac{1}{n}\sum_{j=1}^{n}{YR}_{j}^{2}},\;R_{x}=\sqrt{\frac{1}{n}\sum_{j=1}^{n}{XR}_{j}^{2}},\;R_{t}=\sqrt{\frac{1}{n}\sum_{j=1}^{n}{XR}_{j}^{2}+\frac{1}{n}\sum_{j=1}^{n}{YR}_{j}^{2}}$$

These errors indicate how good the registration is between the input band image and the reference band image. The smaller these errors are, the higher the quality of the four-band multispectral data is. Table 2 indicates that although all of them have a high accuracy, the methods using homography model have a higher precision than those using the polynomial model.

**Table 2:** The errors for registering near infrared, red and green band to the blue band/pixels.

	G-B			G-R			G-IR		
	*R_x_*	*R_y_*	*R_t_*	*R_x_*	*R_y_*	*R_t_*	*R_x_*	*R_y_*	*R_t_*
**R1**	1.17	1.12	1.62	0.98	0.84	1.3	1.7	1.14	2.05
**R2**	1.03	1.21	1.59	1	0.82	1.3	1.52	1.17	1.92
**R3**	1.02	1.14	1.57	0.98	0.8	1.27	1.46	1.09	1.82
**R4**	1.02	1.14	1.57	0.98	0.8	1.27	1.46	1.09	1.82

R1, R2 respectively represents the two methods using the first-order and second-order polynomial model. R3 represents the methods using the homography model. Compared to R3, R4 uses the false matches rejecting method to remove most of the wrong matches first.

### 5.2. Experiment 2

**Figure 14:**
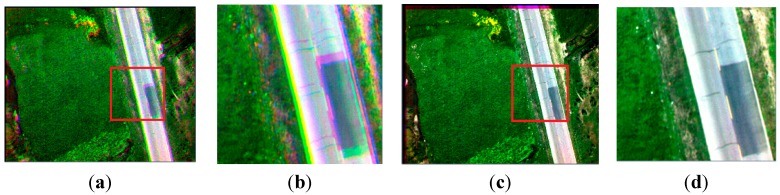
The true-color composite (red, green, blue); (**a**) the unregistered multispectral image; (**b**) the enlarged partial region of (**a**); (**c**) the registered multispectral image; (**d**) the enlarged partial region of (**c**).

**Figure 15:**
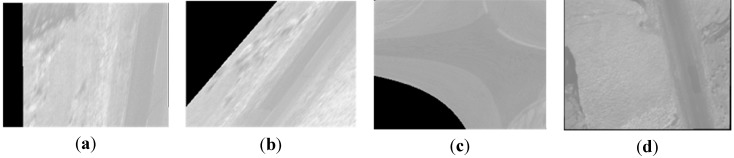
The single infrared bands of the multispectral images; (**a**–**c**) respectively the infrared bands obtained by using the methods using the first-order polynomial, second-order polynomial and homography mode and using the RANSAC directly; (**d**) the infrared band obtained by using our method.

**Figure 16:**
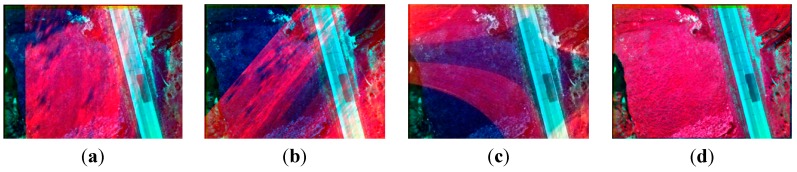
The CIR composite of the four-band images; (**a**–**c**) respectively the multispectral images obtained by using the methods using the first-order polynomial, second-order polynomial and homography model and using the RANSAC directly; (**d**) the multispectral images obtained by using our method.

Compared with Figure 4, nearly all of the ground objects shown in Figure 6 are the grass except a road. The four methods in the previous section are used to compose one four-band multispectral image. Figure 14 depicts the true-color composite and CIR composite. Figure 14a shows the true-color composite of the unregistered multispectral image, and Figure 14b is its enlarged partial region. An obvious dislocation can be seen in Figure 14a,b. Figure 14c shows the registered true-color composite, and Figure 14d is its enlarged partial region. The dislocation is missing. Because of the lack of artificial objects in these images, there is a great difference between visible SIFT features and infrared SIFT features, and the correct rate of initial matching point pairs is significantly lower than 50%. The first three methods use the RANSAC directly; therefore they cannot get a correct infrared band in the four-band multispectral image, as shown in the first three severely distorted images of Figure 15. However, the fourth method uses the rejecting false matches method mentioned in Section 3 to remove most false matches first for promoting the correct rate, and then uses the RANSAC, so it can get a correct infrared band, as shown in the fourth picture of Figure 15. So, the first three methods cannot get a correct CIR composite, as shown in the first three images of Figure 16, but the fourth can, as shown in the fourth image of Figure 16. Table 3, quantitative evaluation of registration error, shows that the errors of the third method and the fourth method are the same, and a little better than the first and second method at column G-B and G-R. At the G-IR column, the first three methods cannot obtain correct results, but the fourth method can get correct result; and its total error, about 2.4 pixels, is still very low.

**Table 3:** The errors for registering near infrared, red and green band to the blue band.

	G-B			G-R			G-IR		
	*R_x_*	*R_y_*	*R_t_*	*R_x_*	*R_y_*	*R_t_*	*R_x_*	*R_y_*	*R_t_*
**R1**	1.03	1.26	1.63	0.83	0.93	1.25	146	309	342
**R2**	1.02	1.27	1.63	0.82	0.93	1.24	328	377	500
**R3**	0.92	1.14	1.48	0.78	0.84	1.16	195	249	295
**R4**	0.92	1.14	1.48	0.78	0.84	1.16	1.3	2.06	2.43

### 5.3. Experiment 3

**Figure 17:**
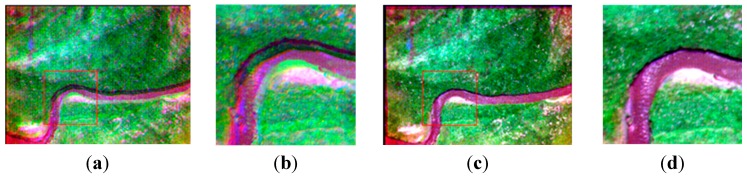
The true-color composite (red, green, blue); (**a**) the unregistered multispectral image; (**b**) the enlarged partial region of (**a**); (**c**) the registered multispectral image; (**d**) the enlarged partial region of (**c**).

**Figure 18:**
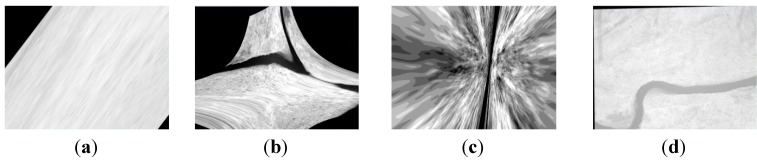
The single infrared band of the multispectral images; (**a**–**c**) respectively the infrared bands obtained by using the methods using the first-order polynomial, second-order polynomial and homography model and using the RANSAC directly; (**d**) the infrared band obtained by using our method.

**Figure 19:**
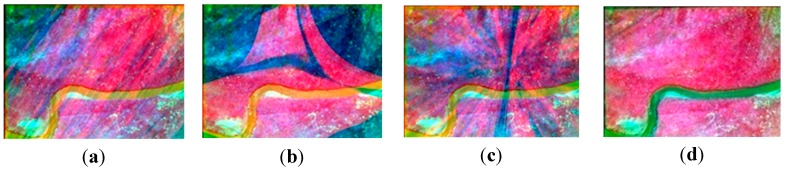
The CIR composite of the four-band images; (**a**–**c**) respectively the multispectral images obtained by using the methods using the first-order polynomial, second-order polynomial and homography model and using the RANSAC directly; (**d**) the multispectral images obtained by using our method.

Compared with Figure 4 and Figure 6, Figure 8 contains no man-made objects. The four methods are used respectively to compose one four-band image. Figure 17a shows the true-color composite of the unregistered multispectral image, and Figure 17b is its enlarged partial region. The dislocation is obvious. Figure 17c,d show the true-color composite of the registered multispectral image. There is no dislocation between different bands. Because there are no artificial objects in these images, there is a great difference between the visible band SIFT features and infrared band SIFT features, and the correct rate of initial matches is lower than 50%, so the first three methods cannot get a correct infrared band in the four-band image, as shown in the first three severely distorted images of Figure 18. Compared with the other three methods, the fourth method uses the rejecting false matches method mentioned in the Section 3 to remove most false matches firstly, and then uses the RANSAC; therefore it can get a correct infrared band, as shown in the fourth image of Figure 18. So, the first three methods cannot get the CIR composite correctly, as shown in the first three images of Figure 19, but the fourth method can, as shown in the fourth image of Figure 19. Table 4 shows that the errors of the third method and the fourth method are the same at the column G-B and G-R, and a little better than the first and second method. At the G-IR column, the first three methods cannot get correct results, but the fourth method can get correct results with a very low error, about 2.5 pixels.

**Table 4:** The errors for registering near infrared, red and green band to the blue band.

	G-B			G-R			G-IR		
	*R_x_*	*R_y_*	*R_t_*	*R_x_*	*R_y_*	*R_t_*	*R_x_*	*R_y_*	*R_t_*
**R1**	1.07	1.31	1.69	0.85	0.97	1.29	227	308	383
**R2**	1.05	1.28	1.66	0.84	0.93	1.25	125	249	279
**R3**	0.95	1.12	1.47	0.83	0.92	1.24	194	237	306
**R4**	0.95	1.12	1.47	0.83	0.92	1.24	1.4	2.08	2.5

## 6. Conclusions

Using four changeable bandpass filters, four identical monochrome cameras, a set of digital video recorders (DVR) and a ruggedized PC, we developed an airborne high resolution multispectral mapping system. The homography registration model was chosen, and we provided a calculation method for its parameters. In the case of fewer or no artificial objects, the conventional SIFT registration approach cannot solve the matching problem between visible images and infrared images. Toward this situation, based on the structural characteristics of the four-camera multispectral system, an effective method to reject most false matches was proposed, and then the RANSAC and the homography model were used to remove the remaining false matches and calculate registration parameters. Experimental results show that for this four-camera multispectral imaging system, the multispectral image creating method can obtain a high quality multispectral image, and band-to-band alignment error is less than 2.5 pixels.
